# Nanoadsorbents for oilfield produced water treatment: synthesis of silica and magnetite nanoparticles for naphthenic acid removal

**DOI:** 10.1007/s11356-026-37530-4

**Published:** 2026-02-21

**Authors:** Juliana Dumard Carracena Candido, João Victor Marques Teixeira, Silvio Edegar Picoli Weschenfelder, Helen Conceição Ferraz

**Affiliations:** 1 Chemical Engineering Program, COPPE, Universidade Federal Do Rio de Janeiro, Rio de Janeiro, Brazil; 2 Escola de Química, Universidade Federal Do Rio de Janeiro, Rio de Janeiro, Brazil; 3 Centro de Pesquisas, Desenvolvimento e Inovação Leopoldo Américo Miguez de Mello, Petrobras, Rio de Janeiro, Brazil

**Keywords:** Silica nanoparticle, Magnetite nanoparticle, Core-shell magnetic nanoparticle, Nanoadsorbent, Naphthenic acid, Oilfield produced water, Water-soluble organics (WSO)

## Abstract

The treatment of produced water, generated in large volumes during oil and gas production, poses a significant environmental challenge faced by the petroleum industry, particularly due to the presence of dissolved organic contaminants such as naphthenic acids. This study reports the synthesis and characterization of nanoadsorbents based on silica and magnetic nanoparticles for application in the polishing stage of produced water treatment, specifically targeting the removal of naphthenic acids. Silica nanoparticles were synthesized using a modified Stöber method, while magnetic nanoparticles were obtained through a coprecipitation method. The surface properties of these nanoparticles were modified to enhance their interaction with naphthenic acids, as confirmed by changes in particle size, as well as their chemical and surface properties. In batch adsorption experiments using synthetic produced water, silica nanoparticles with hydrophobic surface groups exhibited the highest benzoic acid adsorption capacity, reaching 21.9 mg/g. These results highlight the potential of engineered nanomaterials as effective adsorbents, where hydrophobic interactions dominate the adsorption mechanism under the evaluated conditions. An inverse relationship between pH and removal efficiency was observed, and it was demonstrated that the removal efficiency remains consistent across the industrially relevant pH range of 5.5 to 8. Furthermore, the SiO_2_ nanoadsorbent demonstrated a superior multicomponent adsorption capacity, validating the potential of these nanoadsorbents for the treatment of real oilfield produced water.

## Introduction

Produced water treatment is currently one of the major challenges in the oil industry. As the most abundant byproduct of petroleum extraction, produced water is generated at an average global ratio of approximately three volumes of water for every volume of oil (Fakhru’l-Razi et al. 2009; Jiménez et al. 2018). Furthermore, depending on the type and stage of the operation, produced water may account for up to 80% of the total waste generated from oil and gas activities (Neff et al. 2011), emphasizing the importance of addressing this issue.

Produced water has a complex composition, consisting mainly of dispersed and dissolved oil, salts, and chemical additives (Fakhru’l-Razi et al. 2009; Jiménez et al. 2018). Among these components, organic compounds raise the most significant environmental concerns (Neff et al. 2011). As such, they represent the main focus of produced water treatment, especially in cases where the treated water is intended for marine discharge, a common scenario in offshore platforms (Liu et al. 2021b, 2021a).

The discharge of produced water into the sea is regulated by environmental authorities, which set maximum limits for total oil and grease (TOG) and other contaminants. In Brazil and the Gulf of Mexico, for example, the TOG discharge limits are 42 mg/L (daily average) and 29 mg/L (monthly average) (CONAMA. 2007; Venancio et al. 2024). A similar limit is enforced in the North Sea, where the monthly average must not exceed 30 mg/L (Hendges et al. 2021). Additionally, in 2018, Brazilian regulations were updated to include a new method for characterizing produced water designated for marine disposal. With this change, water-soluble organics (WSOs), previously undetected by earlier TOG quantification methods, began to be identified (Neto et al. 2023).

On offshore platforms, produced water is conventionally treated through a series of processes that include three-phase separation (oil, water, and gas), followed by hydrocyclones and flotation units (Walsh and Frankiewicz 2010). While this sequential approach is efficient in removing dispersed oil, it is less effective at eliminating water-soluble organics (WSOs) (Piccioli et al. 2020). Consequently, there is a growing need to develop and implement treatment strategies that effectively remove WSOs and can be integrated with existing conventional treatment systems.

Among all the water-soluble organics found in produced water, naphthenic acids are the most abundant class. Other possible contaminants include BTEX (benzene, toluene, ethylbenzene, and xylene), PAHs (polycyclic aromatic hydrocarbons), and phenols (Ekins et al. 2007; Walsh 2018; Dudek et al. 2020). While naphthenic acids are technically defined as saturated cyclic carboxylic acids, the term is commonly used to refer to a broad range of petroleum-derived organic acids, linear and branched, especially those with more than five carbon atoms (Gruber et al. 2012; Walsh et al. 2014). These compounds exhibit low volatility, high chemical stability, and surfactant-like behavior (Holowenko et al. 2002). As such, both acids and their salts tend to accumulate at the oil–water interface, promoting the stabilization of colloidal systems (Clemente and Fedorak 2005; Gruber et al. 2012; Rodrigues 2018; Simonsen et al. 2019). Therefore, this further reinforces the need for effective treatment methods to remove such compounds.

Naphthenic acids can be effectively removed from produced water using a post-treatment polishing step, following conventional treatment processes. Several techniques are available for this purpose, including advanced oxidation processes, solvent extraction, adsorption, biological treatments, membrane separation, and freeze–thaw evaporation (FTE) (Zheng et al. 2016; Al-Ghouti et al. 2019; Kabyl et al. 2020). Among these, adsorption is particularly attractive due to its effectiveness in removing low-concentration contaminants, operational simplicity, shorter processing times, and lower overall costs compared to alternative technologies (Yousef et al. 2020). Additionally, adsorption offers high versatility, as a wide variety of adsorbent materials can be employed and adapted to the specific properties of the effluent to maximize the removal of organic compounds (Rashed et al. 2020). Numerous adsorbents have been successfully tested for the removal of naphthenic acids from both real and synthetic produced water, including activated carbon and biochars (Martinez-Iglesias et al. 2015; Niasar et al. 2016, 2019; Samrat Alam et al. 2016; Islam et al. 2018; Mônaco et al. 2020; Singh et al. 2020; Lopes et al. 2022; Strong et al. 2023; Medeiros et al. 2023a; Costa Louzada et al. 2023; Machado et al. 2025), commercial adsorbents (Hendges et al. 2021), mesoporous zeolites (Migliorin et al. 2022), and polymers (Vieira et al. 2024).

Nanomaterials have recently gained attention as nanoadsorbents due to their high specific surface area, excellent adsorption performance, efficiency, and potential for reuse (Liang and Esmaeili 2021). Among them, silica nanoparticles stand out for their ability to undergo surface functionalization, which enhances their interaction with target contaminants and improves their adsorption performance (Goh et al. 2019). Similarly, magnetic nanoparticles can be coated with a silica shell to form core–shell nanostructures, which can also be functionalized. The use of magnetic nanomaterials in produced water treatment is regarded as a promising alternative to overcome the operational limitations of conventional adsorbents. Unlike non-magnetic nanoadsorbents, magnetic nanoparticles enable the simple and rapid separation from treated solutions via the application of external magnetic fields (Prigiobbe et al. 2015; Ko et al. 2016).

Regarding the adsorption of naphthenic acids, the surface charge of adsorbents, in combination with the charge of the adsorbate, is known to play a crucial role in the adsorption process. Depending on the nature of these charges, adsorption may occur through different mechanisms, such as electrostatic interactions or hydrophobic forces (Singh et al. 2020). Moreover, other factors, such as the variety of functional groups on the adsorbents, can also affect adsorption behavior. Thus, investigating the influence of surface functionalization on the efficiency and selectivity of adsorption becomes essential.

This study aims to synthesize and comprehensively characterize a variety of nanoadsorbents, including silica-based, magnetic, and core–shell magnetic-silica nanoparticles, as well as their surface-functionalized derivatives. The work also investigates their efficiency in removing naphthenic acids from produced water, with a particular focus on how surface modifications enhance their adsorption capacity and potential application in produced water post-treatment polishing processes.

## Materials and methods

### Materials

All chemicals used in this study were of analytical grade and were used without further purification.

For the synthesis of the silica and magnetite nanoparticles, the following reagents were used: ethanol (99%, Proquimios), cetyltrimethylammonium bromide (CTAB, 98%, Vetec), ammonium hydroxide (28–30%, Vetec), tetraethyl orthosilicate (TEOS, 99%, Sigma Aldrich), (3-aminopropyl)trimethoxysilane (APTMS, 97%, Sigma Aldrich), glacial acetic acid (99.7%, Vetec), methanol (99.8%, Sigma Aldrich), sodium hydroxide (97%, Neon), calcium chloride dihydrate (99%, Proquimios), iron (III) chloride hexahydrate (97%, Sigma Aldrich), and iron (II) sulfate heptahydrate (99%, Proquimios).

For the preparation of the synthetic oilfield produced water, its quantification, and the adsorption tests, the following reagents were used: benzoic acid (99.5%, Sigma Aldrich), cyclohexaneacetic acid (99%, Sigma Aldrich), naphthenic acid (Sigma Aldrich), sodium chloride (99%, Isofar), sodium hydroxide (97%, Neon), hydrochloric acid (37%, Neon), n-hexane (99%, ACS Científica), n-heptane (99.5%, Isofar), and sodium sulfate (anhydrous, Isofar).

### Synthesis of silica nanoparticles

Silica nanoparticles (SiO_2_) were synthesized by the Stöber modified method, and the methodology was adapted from Lanč et al. (2018). First, 400 mL of deionized water and 236 mL of ethanol were mixed in a glass bottle. Then, 7.84 mL of TEOS and 1.4 g of CTAB were added to the mixture, which was left under magnetic stirring until complete solubilization. After that, 7.84 mL of ammonium hydroxide was slowly added to the bottle, causing the precipitation of the silica nanoparticles (SiO_2_). After 21 h, the nanoparticles were collected by centrifugation and washed alternately with deionized water and ethanol until the pH reached neutrality. They were then dried in an oven at 70 °C for 24 h. Unlike traditional template-assisted methods where surfactants are removed, CTAB was deliberately maintained on the nanoparticle surface to serve as permanent functionalization of SiO_2_. Additionally, a CTAB-free nanoparticle was also prepared by calcinating SiO_2_ at 600 °C for 6 h (SiO_2_-c).

### Surface modification of silica nanoparticles

The surface of the calcined silica nanoparticles (SiO_2_-c) was modified by the insertion of –OH groups (SiO_2_-cA) through their resuspension in a 0.01 mol/L sodium hydroxide solution at 60 °C for 24 h, followed by subsequent neutralization with 0.1 mol/L acetic acid. The nanoparticles were then collected by centrifugation, washed three times with methanol, and dried in an oven at 120 °C for 20 h.

Afterward, basic functional groups were inserted on its surface (SiO_2_-cA-NH_2_) using APTMS, following a methodology adapted from Westcott et al. (1998), Lu (2013), and Berktas et al. (2020). One gram of SiO_2_-cA nanoparticles was dispersed in 78 mL of ethanol using ultrasonication. Then, the suspension was transferred to a glass bottle containing 133 mL of deionized water under magnetic stirring. Then, 2.61 mL of ammonium hydroxide and 2 mL of APTMS were added to the mixture, which was kept under magnetic stirring for 24 h. After that, the nanoparticles were collected by centrifugation and washed alternately with deionized water and ethanol until neutral pH and then dried in an oven at 70 °C for 24 h.

### Synthesis of magnetic nanoparticles

Magnetic magnetite nanoparticles (Fe_3_O_4_) were synthesized by the coprecipitation method, following the methodology reported by Bedê et al. (2017). First, a solution of 0.5 mol/L Fe^2+^ was prepared by the reaction between calcium chloride dihydrate and iron (II) sulfate heptahydrate (Eq. 1):

1 $${\mathrm{FeSO}}_{4}.7{\mathrm{H}}_{2}\mathrm{O}+\text{ Ca}{\mathrm{Cl}}_{2}.2{\mathrm{H}}_{2}\text{O }\to {\mathrm{CaSO}}_{4}\downarrow +\text{ Fe}{\mathrm{Cl}}_{2}+9{\mathrm{H}}_{2}\mathrm{O}$$

Then, 100 mL of the 0.5 mol/L FeCl_2_ solution was added to a jacketed borosilicate glass reactor and mixed with 100 mL of a 0.5 mol/L FeCl_3_ solution, prepared by dissolving iron (III) chloride hexahydrate in deionized water. The resulting solution was mixed by mechanical stirring and heated to 60 °C. Afterwards, 25 mL of ammonium hydroxide was added to the reactor, resulting in the instant formation of a black precipitate (Fe_3_O_4_) (Eq. 2):

2 $${\mathrm{FeCl}}_{2}+2{\mathrm{FeCl}}_{3}+8{\mathrm{NH}}_{4}\mathrm{OH}\to {\mathrm{Fe}}_{3}{\mathrm{O}}_{4}\downarrow + 8{\mathrm{NH}}_{4}\mathrm{Cl}+4{\mathrm{H}}_{2}\mathrm{O}$$

The reaction was allowed to proceed for 15 min and then cooled to room temperature. Finally, the magnetite nanoparticles were collected by a magnet, washed until neutral pH with deionized water, and then oven-dried at 70 °C for 18 h.

### Surface modification of magnetic nanoparticles

Core–shell magnetic nanoparticles (Fe_3_O_4_@SiO_2_) were prepared using a methodology adapted from Elmobarak and Almomani (2021). First, 500 mg of Fe_3_O_4_ nanoparticles were dispersed in 25 mL of deionized water under ultrasonication. The suspension was transferred to a glass bottle containing 100 mL of ethanol, under mechanical stirring. Then, 1 mL of ammonium hydroxide and 95 mg of CTAB were added to the mixture. After homogenization, 500 µL of TEOS was added, and the system was kept under mechanical stirring for 21 h. After that, Fe_3_O_4_@SiO_2_ core–shell nanoparticles were collected using a magnet, washed alternately until neutral pH with deionized water and ethanol, and oven-dried at 70 °C for 24 h.

Core–shell magnetic nanoparticles were further modified by the insertion of amino groups into their surface, following a methodology adapted from Westcott et al. (1998) and Berktas et al. (2020). 500 mg of Fe_3_O_4_@SiO_2_ core–shell nanoparticles were suspended in 39 mL of ethanol by sonication. Then, the suspension was transferred to a glass bottle containing 66.5 mL of deionized water, under mechanical stirring. After that, 1.3 mL of ammonium hydroxide and 1 mL of APTMS were added. After 24 h, the functionalized core–shell nanoparticles (Fe_3_O_4_@SiO_2_-NH_2_) were collected using a magnet, washed alternately with deionized water and ethanol until neutral pH, and oven-dried at 70 °C for 24 h.

**Fig. 1:**
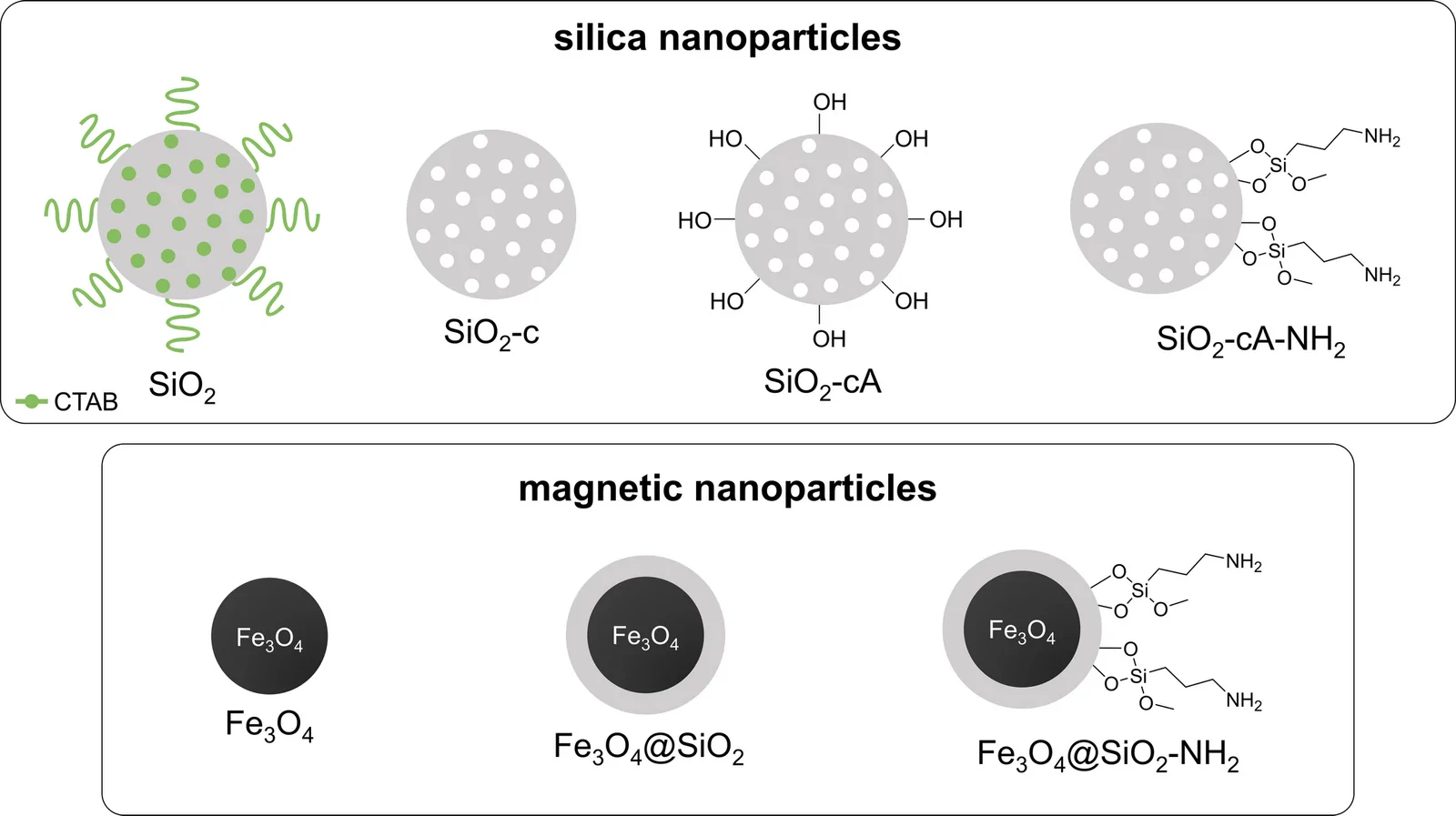
Schematic representation of silica and magnetic nanoparticles. A green circle represents CTAB imprisoned in the pores of SiO_2_ nanoparticles, and the empty pores in the nanoparticles are represented by white circles

Figure 1 shows the schematic representation of the nanoparticles synthesized in this study.

### Nanoparticle characterization

The nanoparticles were characterized by the following techniques and equipment: elemental analysis (CHNS)—Thermo Scientific FlashSmart CHNS/O (ISI B&F–SENAI/CETIQT); thermogravimetric analysis (TGA)—Hitachi STA 7300 (ISI B&F–SENAI/CETIQT); Fourier transform infrared spectroscopy (FTIR)—PerkinElmer Frontier FT-IR/FIR (LAPIN1–IMA/UFRJ); dynamic light scattering (DLS) and zeta potential—Anton Paar Litesizer 500 (LABEFIT – PEQ/COPPE/UFRJ); textural analysis—MicrotracBel BELCAT II (ISI B&F–SENAI/CETIQT); scanning electron microscopy (SEM)–Tescan MIRA 4th generation LMU (IMA/UFRJ); and transmission electron microscopy (TEM)—FEI Tecnai G^2^ Spirit BioTWIN (CEATIM–INMETRO).

### Batch adsorption studies

To evaluate the adsorption capacity of the synthesized nanoparticles, synthetic solutions of produced water, containing model naphthenic acids, were prepared according to the methodology described by de Carvalho Neto et al. (2022) and adapted from Iranmanesh et al. (2014). Benzoic acid was chosen as a model naphthenic acid due to its high solubility in water, which makes it difficult to remove from produced water (Frankel et al. 2016; Bergfors et al. 2021). Cyclohexaneacetic acid was chosen as a comparative naphthenic acid, as it is an alicyclic carboxylic acid. Finally, a multicomponent synthetic produced water was also tested, using a commercial mixture of naphthenic acids. The synthetic produced water was prepared by dissolving sodium chloride (100 g/L), sodium hydroxide (0.05 mol/L), and benzoic acid (250 mg/L), or cyclohexaneacetic acid (100 mg/L) in deionized water, under magnetic stirring for 72 h. Sodium hydroxide was used to increase the pH and deprotonate the benzoic acid, thus increasing its solubility in water. The multicomponent synthetic produced water, prepared with the naphthenic acid mixture, was homogenized by ultrasonication for 30 min instead of magnetic stirring and was filtered to remove any dispersed fraction.

Preliminary kinetic experiments were performed to determine the time required for the system to reach adsorption equilibrium. It was observed that the adsorption capacity reached a plateau in less than 24 h. Consequently, a contact time of 24 h was adopted for all subsequent batch experiments to ensure that all reported data represent equilibrium conditions.

The adsorption capacity of the nanoparticles was evaluated through batch experiments, where 50 mL of the synthetic produced water had its pH adjusted and was agitated at 180 rpm in a shaker incubator at 30 °C. After temperature equilibration, 200 mg of nanoparticles was added, and after 24 h, the liquid and solid phases were separated by centrifugation. Then, UV spectroscopy (Agilent BioTek Epoch) was used to quantify the concentration of benzoic acid in the resulting solution, using a pre-prepared calibration curve. The residual concentration of cyclohexaneacetic acid and the mixture of naphthenic acids was determined by an indirect method, in which the samples were extracted with n-heptane or n-hexane and then analyzed by FTIR (Shimadzu IRTracer-100).

### Influence of acid type and pH on the adsorption process

A two-factor factorial design (2 × 3) with a continuous pH factor and a categorical acid-type factor, with replicated center points on pH, was carried out to determine the influence of pH and the structure of the naphthenic acid in the adsorption process. Benzoic acid and cyclohexaneacetic acid were studied as naphthenic acid models. pH was chosen according to the pKa of the model acids and the pI of the nanoadsorbent. Table 1 shows the list of experiments, executed in random order. For these experiments, the SiO_2_ nanoparticle was chosen as the adsorbent, as it had the best adsorption performance of benzoic acid at pH 3. To evaluate the significance of the factors, Eq. 3 was fitted to the data.

3 $$y= {\beta }_{0}+{\beta }_{1}{x}_{1}+{\beta }_{2}{x}_{2}+{\beta }_{12}{x}_{1}{x}_{2}+{\beta }_{22}{x}_{2}^{2}+\varepsilon$$

where *y* is the predicted response (removal efficiency), *x*_*1*_ and *x*_*2*_ are the coded values for acid type and pH, respectively, *β*_*0*_, *β*_1_, *β*_*2*_, *β*_*12*_, and *β*_*22*_ are the regression coefficients, and *ε* is the random error.

**Table 1:** Two-factor factorial design (2 × 3) with triplicate central point on pH

Experiment	Acid	pH
1	− (benzoic)	− (3)
2	− (benzoic)	0 (5.5)
3	− (benzoic)	+ (8)
4	+ (cyclohexaneacetic)	− (3)
5	+ (cyclohexaneacetic)	0 (5.5)
6	+ (cyclohexaneacetic)	+ (8)
7	− (benzoic)	0 (5.5)
8	− (benzoic)	0 (5.5)
9	+ (cyclohexaneacetic)	0 (5.5)
10	+ (cyclohexaneacetic)	0 (5.5)

## Results and discussion

### Elemental analysis (CHNS)

Elemental analysis of the silica nanoparticles (Table 2) was performed to confirm the removal of the surfactant from the samples and to verify the functionalization with APTMS.

**Table 2:** Elemental analysis of the silica samples: SiO_2_, SiO_2_-c, SiO_2_-cA, and SiO_2_-cA-NH_2_

Sample	Element (%)			
	**N**	**C**	**H**	**S**
SiO_2_	1.49	23.68	5.48	0
SiO_2_-c	0	0	2.33	0
SiO_2_-cA	0.01	0.07	1.24	0
SiO_2_-cA-NH_2_	0.87	2.69	1.32	0

The results of the elemental analysis for the SiO_2_ nanoparticles showed the presence of nitrogen and a large amount of carbon, indicating the presence of CTAB, used as a porosity-generating and functionalizing agent. This result is different from that expected and observed by other authors, for example, Rafigh and Heydarinasab (2017), who observed the presence of only 0.75% of carbon atoms and the absence of nitrogen atoms in a sample of SiO_2_ nanoparticles, synthesized by the same method and using Tween 80 as a surfactant. Other authors (Wang et al. 2022) used CTAB as a surfactant in the synthesis of silica nanoparticles and obtained similar results to those of the present study, with the presence of 4.62% carbon atoms and 0.22% nitrogen atoms in their samples. Although present, these atoms are found in smaller quantities, which can be justified by the difference in washing the nanoparticles after their synthesis, which was carried out by these authors using methanol and water, rather than ethanol and water. Based on these findings, the alternating water and ethanol washing procedure proved ineffective in eliminating CTAB from the pores of the nanoparticles. This surfactant (cetyltrimethylammonium bromide, C_19_H_42_BrN) features a long hydrocarbon chain and a nitrogen atom in its molecular structure. Thus, it can be concluded that the CTAB functionalization was effective, as the molecule was successfully retained on the nanoparticle surface.

The results obtained for the calcined sample (SiO_2_-c) demonstrate that nitrogen and carbon atoms are no longer present in the material after calcination. This thermal treatment was performed at a temperature exceeding the degradation point of the CTAB molecule (Putz et al. 2015; Kunc et al. 2019), thereby ensuring the removal of surfactant molecules confined within the nanoparticle pores. Additionally, the presence of hydrogen is observed in the SiO_2_-c sample. This can be attributed to the adsorption of water molecules from the surrounding environment. As the surfactant is desorbed during calcination, the pores become available, increasing the specific surface area and adsorption capacity of the material. Consequently, water molecules from the environment are readily adsorbed. This behavior is further supported by the significant mass loss observed at low temperatures in the TGA analysis.

With the surface activation of the SiO_2_-cA sample, part of the surface becomes coated with –OH groups, which would be expected to increase the proportion of hydrogen atoms in the sample. However, elemental analysis results for this sample show a decrease in the hydrogen content compared to the calcined sample (SiO_2_-c). This may be explained by a lower degree of ambient water adsorption in the activated sample. The presence of surface –OH groups hinders water adsorption by introducing steric effects that prevent water molecules from binding effectively to the nanoparticle surface. This reduction in adsorbed water is also supported by the TGA analysis. Therefore, despite the expected increase in hydrogen content due to surface activation, the CHNS analysis indicates a decrease in the hydrogen proportion when compared to the calcined sample.

**Fig. 2:**
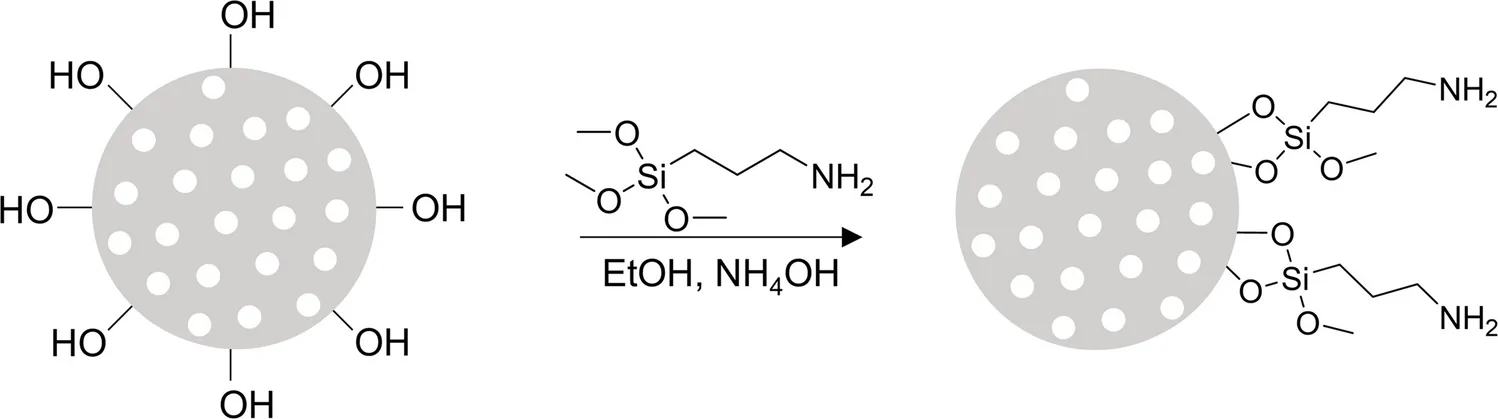
Schematic illustration of the functionalization reaction of activated silica nanoparticles (SiO_2_-cA) with APTMS, yielding functionalized silica nanoparticles (SiO_2_-cA-NH_2_)

The elemental analysis of the functionalized sample (SiO_2_-cA-NH_2_) reveals an increase in both nitrogen and carbon contents relative to the activated sample (SiO_2_-cA). This increase is attributed to the presence of APTMS (Beagan et al. 2022). Furthermore, a nitrogen-to-carbon ratio (N/C) of 0.32 is observed, providing additional evidence of successful functionalization. The functionalization reaction results in nanoparticles grafted with aminopropyl groups (Fig. 2), which are expected to have an N/C ratio of 0.33, closely matching the value measured in the functionalized material.

### Thermogravimetric analysis (TGA)

**Fig. 3:**
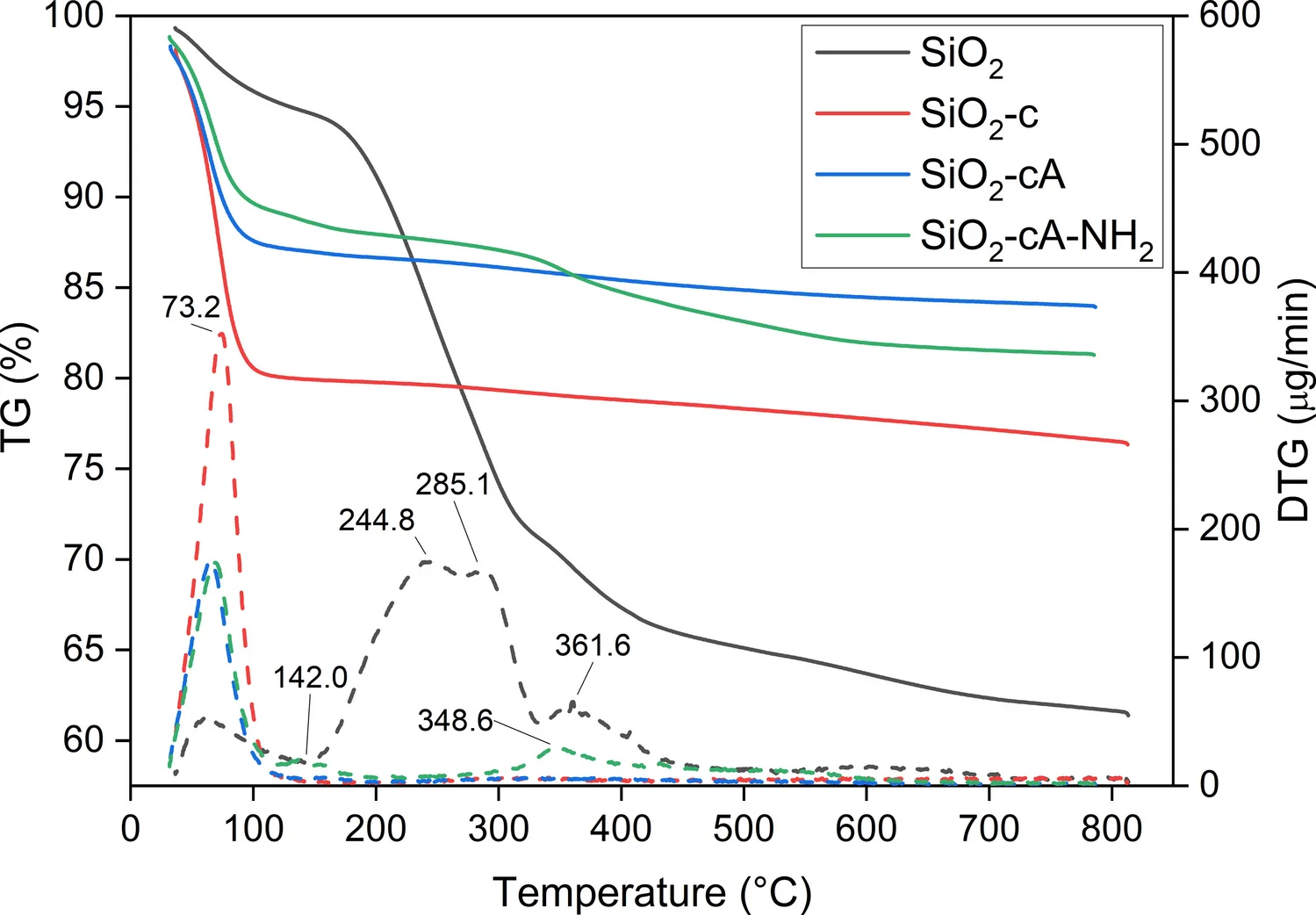
Thermogravimetric analysis curves and their derivatives for silica samples

Thermogravimetric analysis of silica nanoparticles (Fig. 3) was performed to verify CTAB removal and surface modification. The first degradation step observed in all samples, at 73 °C, is attributed to the dehydration of the samples (Lu 2013).

CTAB removal by calcination is verified by the mass loss between 150 and 320 °C in the SiO_2_ sample, which is absent in the SiO_2_-c sample. These two steps correspond to the degradation of the alkyl chain and the polar head of the surfactant, respectively. This mass loss step can also be associated with the dehydroxylation of the silica’s silanol groups (Putz et al. 2015). Therefore, a surface activation step is necessary before functionalization with amino groups. Finally, a last degradation step is observed for the SiO_2_ sample, between 330 °C and 460 °C. This stage is related to the removal of ammonia, used as a synthesis catalyst, which is trapped within the nanoparticle pores (Kunc et al. 2019).

In contrast to the thermogram of the SiO_2_ sample, the thermogram of the calcined sample (SiO_2_-c) exhibits only one degradation step. As the sample underwent thermal treatment at 600 °C, the degradation stages previously described for the SiO_2_ sample occur during calcination and are therefore not present in the thermogram. On the other hand, a sharp mass loss event with a well-defined peak is observed between 40 °C and 110 °C. This mass loss stage is associated with sample dehydration through the desorption of water molecules adsorbed in the nanoparticle pores (Lu 2013). As confirmed by the elemental analysis results, after calcination, the pores of the SiO_2_-c sample, previously filled with surfactant molecules and excess reagents (SiO_2_ sample), are emptied, becoming available for the adsorption of environmental water molecules.

The activation of the SiO_2_-cA sample’s surface introduces –OH groups, which hinder the adsorption of water from the environment. The activation can be verified by the decrease in the degradation peak related to water removal, compared to the SiO_2_-c sample (Lu 2013). The functionalization of the SiO_2_-cA-NH_2_ sample is confirmed by the mass loss between 350 and 600 °C related to the degradation of the aminopropyl. Additionally, the difference in residual mass between this functionalized sample and the unfunctionalized sample further supports this finding (Ruiz-Cañas et al. 2020).

### Fourier transform infrared spectroscopy (FTIR)

**Fig. 4:**
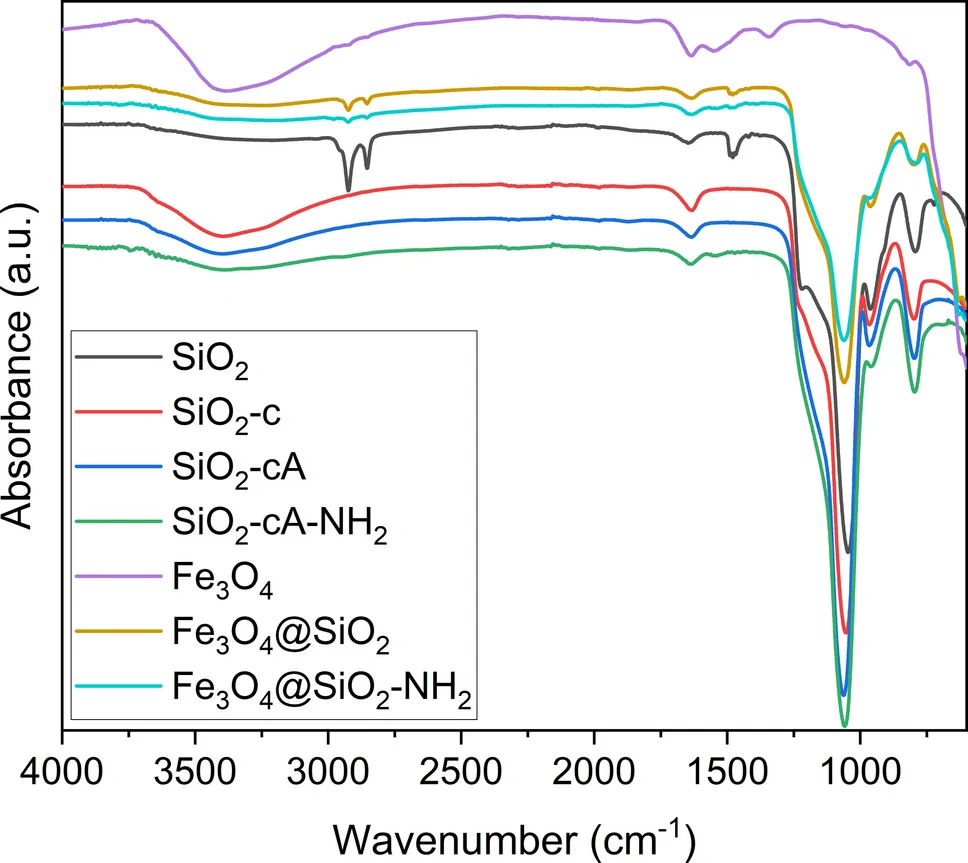
FTIR spectra for silica and magnetite nanoparticles

FTIR analysis (Fig. 4, Table 3) revealed the presence of silica-related bonds in all silica nanoparticles and the core–shell magnetite nanoparticles, with characteristic peaks at 1050, 965, and 800 cm^−1^ (Putz et al. 2015). This indicates the success of the encapsulation. In the magnetic samples, characteristic bonds of magnetite were observed at 620 and 600 cm^−1^ (Asab et al. 2020).

**Table 3:** FTIR spectral bands corresponding to the chemical bonds present in the silica and magnetite nanoparticle samples, along with the characteristic bonds of CTAB and APTMS, the reagents employed in the synthesis processes

**Silica**	
Asymmetric Si–O-Si stretching	1050 cm^−1^
Si–OH vibration	965 cm^−1^
Symmetric Si–O-Si stretching	800 cm^−1^
Si–OH vibration	3700–3000 cm^−1^
Presence of H_2_O	1640 cm^−1^
**Magnetite**	
Fe–O-Fe vibration	620 cm^−1^
Fe–O vibration	~ 600 cm^−1^
Fe-OH stretching	3376 cm^−1^
Presence of H_2_O	1633 cm^−1^
**CTAB**	
Asymmetric -CH_2_ stretching	2924 cm^−1^
Symmetric -CH_2_ stretching	2854 cm^−1^
-CH stretching	1480 cm^−1^
**APTMS**	
-NH_2_ vibration	1600 and 1460 cm^−1^

In addition to the characteristic silica bonds, the FTIR spectrum of the SiO_2_ sample shows additional bands at 2924, 2854, and 1480 cm^−1^, which are attributed to the presence of the surfactant (Alswieleh et al. 2019). These bands are no longer observed after calcination, confirming the complete elimination of the surfactant (Putz et al. 2015), as also supported by the thermal analysis results.

Due to the low intensity of the signals and the overlapping of bands, it was not possible to confirm the functionalization through FTIR. A similar result was reported by Berktas et al. (2020), who were unable to detect the band corresponding to the –NH group in their functionalized samples and concluded that this was due to overlap with the dominant silica band. Nevertheless, they observed evidence of functionalization through the presence of bands at 1600 and 1500 cm^−1^, corresponding to the vibrational modes of the –NH_2_ group, which suggests the presence of terminal groups from the APTES reagent employed in their study.

Regarding the core–shell magnetite nanoparticles, a reduction in the intensity of the iron-related absorption bands at 623 and approximately 600 cm^−1^ is observed. According to Gemeay et al. (2020), this finding provides additional evidence of successful silica shell formation around the magnetic nanoparticles. The silica-coated and functionalized sample, Fe_3_O_4_@SiO_2_-NH_2_, exhibits the same spectral features as the non-functionalized coated sample. Therefore, as with the functionalized silica samples, FTIR analysis alone was not sufficient to confirm successful functionalization with APTMS.

### Zeta potential

**Fig. 5:**
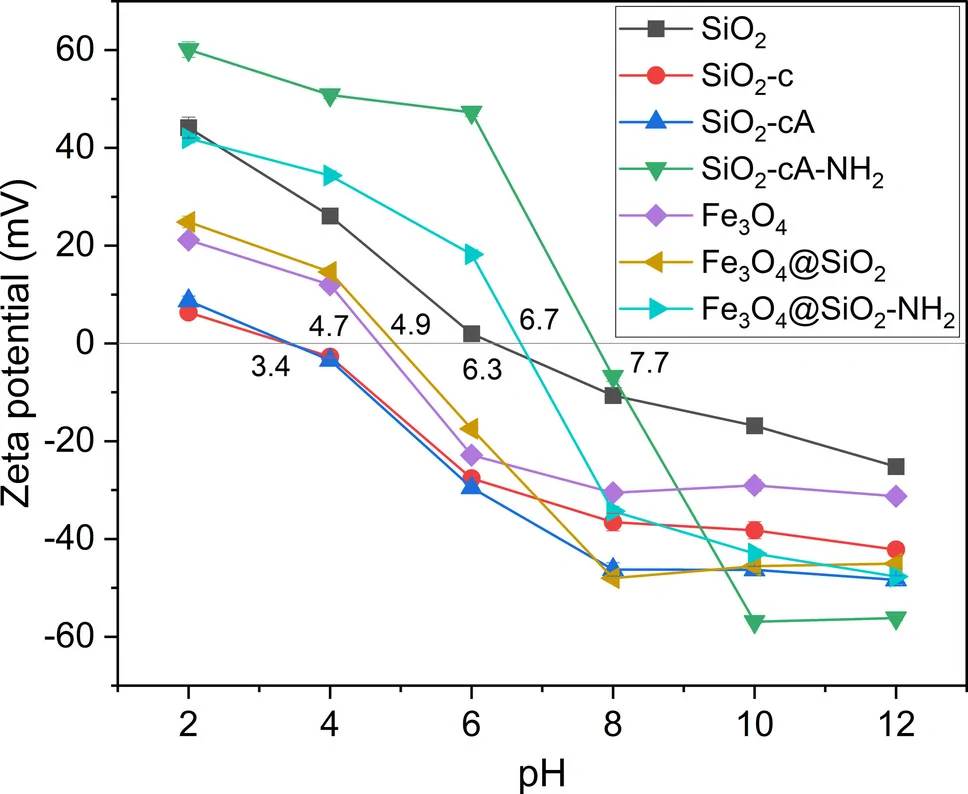
Zeta potential for silica and magnetite nanoparticles

The electrokinetic potential was used to evaluate the functionalization of the nanoparticles, through the correlation between their zeta potential and their surface properties (Fig. 5, Table 4). The SiO_2_ sample has a higher isoelectric point (6.3) than expected for silica (2.0) (Corredor et al. 2018). The elevated zeta potential of the SiO_2_ sample is likely due to the presence of surfactant molecules retained within the porous framework and surface of the nanoparticles. As confirmed by both elemental analysis and FTIR, the sequential washing with ethanol and water was not sufficient to ensure the complete removal of CTAB. As a cationic surfactant, CTAB has a significant impact on the surface chemistry of nanoparticles, resulting in a positive surface charge across a broad pH range. A similar phenomenon was observed by Soares et al. (2019), who evaluated different silica-coating methods for magnetite nanoparticles. The procedure involving CTAB resulted in nanoparticles with a higher isoelectric point than those produced without CTAB, which the authors attributed to the presence of trimethylammonium groups on the nanoparticle surface.

**Table 4:** Isoelectric points, hydrodynamic diameters, and textural properties for the silica and magnetite samples: specific surface area (*S*_*BET*_), BET constant (*C*), monolayer volume (*V*_*m*_), pore diameter (*D*_*p*_), and pore volume (*V*_*p*_)

Sample	Isoelectric point (mV)	Hydrodynamic diameter (nm)	*S*_*BET*_ (m^2^/g)	*C*	*V*_*m*_ (cm^3^/g)	*D*_*p*_ (nm)	*V*_*p*_ (cm^3^/g)
SiO_2_	6.3	559.7 ± 119.8	30	60	7	7.8	0.06
SiO_2_-c	3.4	542.6 ± 79.9	835	177	192	5.5	0.91
SiO_2_-cA	3.4	519.4 ± 76.9	319	165	73	4.2	0.57
SiO_2_-cA-NH_2_	7.7	607.5 ± 96.2	118	78	27	4.2	0.48
Fe_3_O_4_	4.7	40.5 ± 7.7	39 ^a^	-	-	-	-
Fe_3_O_4_@SiO_2_	4.9	60.3 ± 1.3	115	38	26	12.1	0.20
Fe_3_O_4_@SiO_2_-NH_2_	6.7	57.8 ± 1.6	49	53	11	12.1	0.17

^a^The specific surface area of the Fe_3_O_4_ sample was estimated based on the average particle diameter obtained from the SEM analysis, assuming the nanoparticles to be non-porous spheres

With the removal of CTAB, after calcination and activation, the isoelectric point of the SiO_2_-c sample decreases to 3.4. Through surface activation, –OH groups were introduced onto the surface of the SiO_2_-cA nanoparticles, as evidenced by the TGA and CHNS data. However, this modification was not detected in the zeta potential measurements, where the activated sample showed an isoelectric point of 3.4, identical to that of the calcined sample. Nevertheless, the activation process contributed to enhanced colloidal stability, as inferred from the higher absolute values of zeta potential under alkaline pH conditions (Vazquez et al. 2017).

For the magnetic nanoparticles, the zeta potential analysis yielded results consistent with those reported in the literature. The Fe_3_O_4_ sample exhibited an isoelectric point of 4.7. This value is comparable to the pI of 4.86 reported by Liu et al. (2015), who synthesized magnetite nanoparticles using a methodology similar to that employed in the present study. However, this value is slightly lower than those reported by other authors (Wang et al. 2010; Vikesland et al. 2016), which may be attributed to surface oxidation (Soares et al. 2019).

The silica-coated magnetite nanoparticles, Fe_3_O_4_@SiO_2_, exhibited an isoelectric point of 4.9, which lies between the values observed for Fe_3_O_4_ and SiO_2_ nanoparticles. For this sample, an isoelectric point similar to that of the silica SiO_2_ nanoparticle would be expected. FTIR analysis of this sample confirmed the absence of CTAB, which justifies the lower isoelectric point compared to the SiO_2_ sample. Nevertheless, the behavior observed for this sample differs from that reported by other authors, who found isoelectric points around 2, consistent with pure silica nanoparticles (Sun et al. 2005; Wang et al. 2010; Zou et al. 2014; Su et al. 2018). This discrepancy may be attributed to the amount of TEOS used during the coating process, which could have resulted in a thin silica shell surrounding the magnetite nanoparticles. According to Singh et al. (2012), the thickness of the silica shell can influence the zeta potential behavior of silica-coated magnetite nanoparticles; the thicker the silica layer, the more closely the coated nanoparticles resemble the surface behavior of pure silica.

Regarding the surface modifications, the successful functionalization of the SiO_2_-cA-NH_2_ sample can be verified by the increase in its isoelectric point to 7.7 (Schiestel et al. 2004). The same behavior was observed for the magnetic nanoparticles, with an increase in the isoelectric point from 4.9 to 6.7 after functionalization (Wang et al. 2020).

In addition to information regarding the isoelectric point of the nanoparticles, zeta potential analysis as a function of pH can provide insights into their stability in aqueous media. Higher absolute zeta potential values indicate a stronger propensity for particle–particle repulsion, thereby enhancing stabilization and mitigating agglomeration (Vazquez et al. 2017). This information can be useful when selecting the ideal nanoadsorbents for use at specific pH levels during adsorption assays. For instance, according to the results, at acidic pH, the samples with the highest stability are SiO_2_-cA-NH_2_ and Fe_3_O_4_@SiO_2_-NH_2_, whereas at basic pH, the samples with the lowest tendency toward agglomeration are SiO_2_-cA-NH_2_ and SiO_2_-cA.

### Dynamic light scattering (DLS)

The hydrodynamic diameters of the nanoparticles were obtained by DLS (Table 4). All silica nanoparticles presented hydrodynamic diameters of approximately 500–600 nm, as observed by other authors (Lanč et al. 2018). While no statistically significant differences were observed among the hydrodynamic diameters of the silica nanoparticles, the functionalized sample, SiO_2_-cA-NH_2_, showed a slightly larger average diameter than its non-functionalized counterpart, which may indicate successful functionalization. This slight increase in hydrodynamic diameter may be attributed to the anchoring of functional groups on the particle surfaces. However, given the relatively small size of these groups, their contribution to the overall diameter of particles approximately 600 nm in size would be minimal.

On the other hand, the magnetic nanoparticles presented a change in hydrodynamic diameter from 40 to 60 nm after encapsulation in silica. The 20 nm difference between coated and uncoated nanoparticles suggests the formation of a silica shell with an estimated thickness of 10 nm. Thus, it can be concluded that the silica coating procedure was effective.

In addition, the encapsulation of magnetic nanoparticles within a silica shell enhances their aqueous stability by mitigating agglomeration. This phenomenon is attributed to the attenuation of attractive forces and the reduction of inter-particle dipole interactions (Santos et al. 2021). With the coating, these particles are stabilized by reducing the magnetic interaction force, which is partially blocked by the silica layer (Sun et al. 2005). Moreover, the Hamaker constant of silica in water is lower than that of magnetite, indicating weaker attractive forces between the coated nanoparticles and, consequently, a reduction in agglomeration (Faure et al. 2011). The enhanced stability of the silica-coated nanoparticles is reflected in the smaller standard deviation of the hydrodynamic diameter measurements compared to bare magnetic nanoparticles (Table 4).

### Textural analysis (BET)

**Fig. 6:**
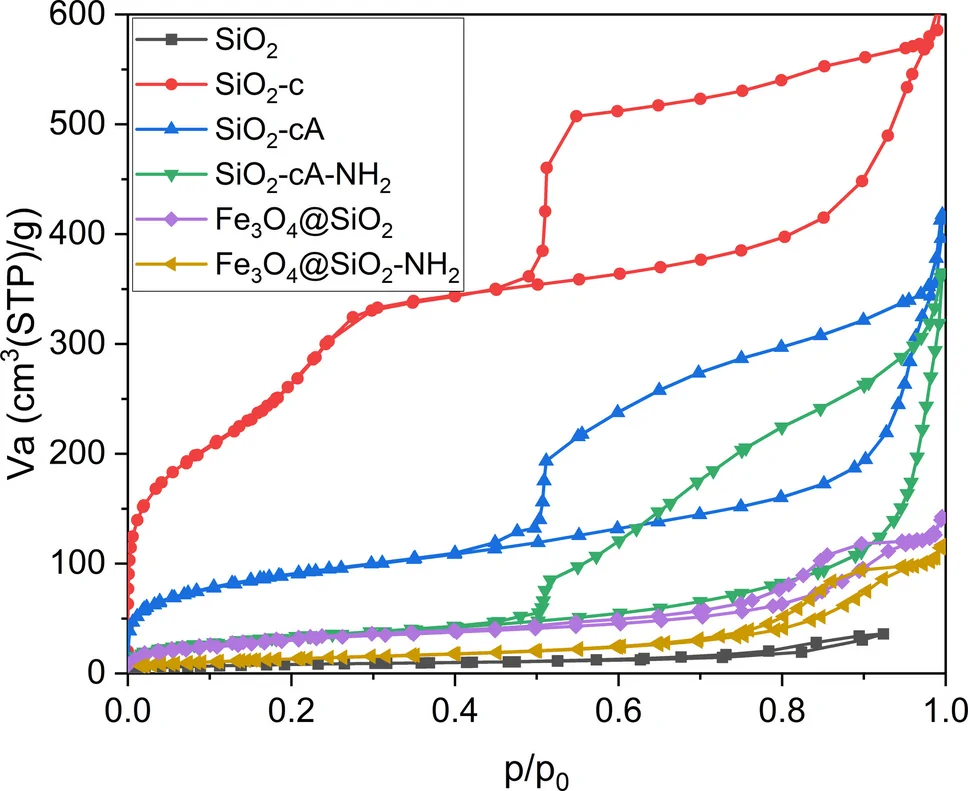
N_2_ adsorption and desorption isotherms at 77 K for the silica and magnetic samples

The nitrogen adsorption isotherms at 77 K of the silica and encapsulated magnetite samples are classified as Type IV, characteristic of mesoporous materials (Fig. 6). With the calcination of the silica sample, there was an increase in the specific surface area from 30 to 835 m^2^/g. With activation and functionalization, this area decreased to 319 and 118 m^2^/g, respectively. Similarly, there was a decrease in the specific surface after functionalization of the Fe_3_O_4_@SiO_2_ sample, from 115 to 49 m^2^/g, due to the insertion of functional groups on both the internal and external surfaces of the nanoparticles (Rafigh and Heydarinasab 2017).

Although all samples exhibit Type IV isotherms, the hysteresis loops and textural properties differ between them. The SiO_2_ sample showed H3-type hysteresis loops, which are typically associated with macroporosity (Thommes et al. 2015). Although mesopores are present in this material, they are likely obstructed by CTAB. As previously discussed, the applied washing and drying methods were insufficient to entirely eliminate the surfactant from the nanoparticle pores. As a result, the mesopores are blocked, and only the larger pores, where partial surfactant removal occurred, contribute to the pore structure. This interpretation is consistent with the low specific surface areas reported for these samples (Table 4). Alternatively, the H3 hysteresis may be indicative of slit-like pore geometries (Condon 2006).

The isotherm of the SiO_2_-c sample exhibits a sharp increase in the adsorbed volume at low relative pressure. This characteristic has been reported by other authors and may be attributed to the presence of micropores in the sample (Bchellaoui et al. 2017; Alotaibi et al. 2021). This interpretation is further supported by the high value of the BET C constant, which was 177 for this sample. Elevated values of this parameter (> 150) are commonly associated with the presence of micropores (Thommes et al. 2015). Upon surface activation with the –OH groups, leading to the SiO_2_-cA sample, the material’s structure is altered, and the initial isotherm behavior changes accordingly. The micropores become filled, and the specific surface area decreases from 835 to 319 m^2^/g. A similar trend is observed for the SiO_2_-cA-NH_2_ sample, in which the specific surface area further decreases following functionalization.

The average pore size presented by the SiO_2_-c sample is smaller than that of the non-calcined samples, as shown in Table 4. Consequently, there is also an increase in the total pore volume of the calcined sample. The same behavior was observed by Putz et al. (2015), who reported a decrease in pore size and an increase of approximately 10 to 25 times in pore volume of silica samples prepared with CTAB and catalyzed with ammonia after calcination at 700 °C. The addition of CTAB, followed by its complete removal during calcination, is also responsible for the increase in the specific surface area of the SiO_2_-c sample. Furthermore, as noted by Vazquez et al. (2017), the use of CTAB in silica nanoparticle synthesis yields materials with a greater number of interconnected pores, which enhances the surface area. Upon surface activation and functionalization (samples SiO_2_-cA and SiO_2_-cA-NH_2_), both average pore size and pore volume are reduced, a result attributed to the incorporation of functional groups on the internal and external surfaces of the nanoparticles (Rafigh and Heydarinasab 2017).

Regarding the magnetite samples encapsulated in silica, H3-type hysteresis loops are observed, similar to those seen for the SiO_2_ sample. The same behavior was reported by Gemeay et al. (2020). Despite the similarity to the previously mentioned sample, the specific surface area of the Fe_3_O_4_@SiO_2_ sample was about three times greater than that of the silica, suggesting effective removal of the surfactant used during synthesis through the washing process. Comparing the two encapsulated magnetic nanoparticles, a reduction in specific surface area following functionalization is revealed, consistent with the trend observed between SiO_2_-cA and SiO_2_-cA-NH_2_ samples, likely due to the incorporation of functional groups on both the internal and external surfaces of the nanomaterials (Rafigh and Heydarinasab 2017).

### Scanning and transmission electron microscopy (SEM and TEM)

**Fig. 7:**
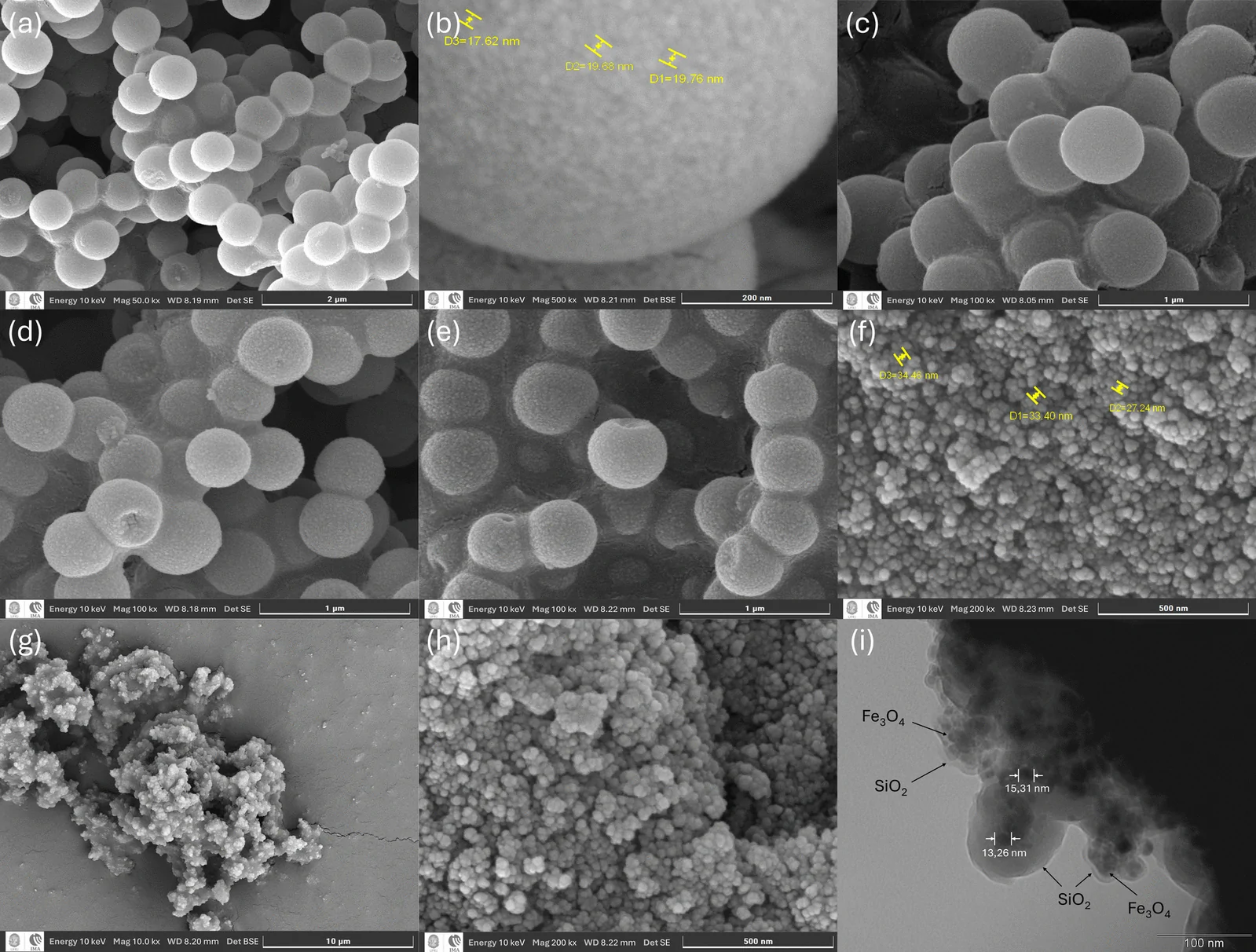
SEM images of the samples: **a, b** SiO_2_; **c** SiO_2_-c; **d** SiO_2_-cA; **e** SiO_2_-cA-NH_2_; **f** Fe_3_O_4_; **g** Fe_3_O_4_@SiO_2_; and **h** Fe_3_O_4_@SiO_2_-NH_2_; along with **i** a TEM image of the Fe_3_O_4_@SiO_2_ sample. The corresponding scale bars in the images are 2 µm, 200 nm, 1 µm, 1 µm, 1 µm, 500 nm, 10 µm, 500 nm, and 100 nm, respectively

SEM and TEM images of the samples are shown in Fig. 7. All silica nanoparticles exhibited a spherical shape and homogeneous size distribution, as observed by other authors (Wang et al. 2012; Hernandez-Leon et al. 2017). The SiO_2_ sample had a diameter of about 600 to 700 nm. The surface roughness of this sample (~ 19 nm) was attributed to CTAB micelles used as a surfactant during synthesis (Patel et al. 2014), which were not completely removed by washing, requiring calcination for complete removal.

After calcination, the size of the nanoparticles decreased to approximately 500–600 nm, indicating structural consolidation of the silica (Putz et al. 2015). Functionalization led to increased surface roughness due to the attachment of functional groups (Wang et al. 2012), though some surface degradation occurred in the functionalized sample, possibly from ultrasonication.

Magnetic Fe_3_O_4_ nanoparticles also showed spherical shapes with diameters near 30 nm (Fig. 7), similar to that observed by Ma et al. (2012). According to the SEM and TEM images, the Fe_3_O_4_@SiO_2_ nanoparticles had spherical morphology with multiple magnetite cores (~ 15 nm) encapsulated by a silica shell (Camacho-Fernández et al. 2021), but their tendency to form large agglomerates made it difficult to observe individual nanoparticles.

This tendency toward agglomeration can decrease the area available for adsorption due to the reduction of the reactive surface. However, in applications where the collection of nanoparticles via magnetic fields is required for real-time separation, such as the one proposed in this study, magnetic nanoparticles’ agglomeration may not represent a disadvantage. When magnetic nanoparticles assemble into clusters within the nanometer-to-micrometer scale, their recovery is facilitated because the magnetic effect is a cooperative phenomenon (Lim et al. 2013).

### Adsorption of a model naphthenic acid

The adsorption of a model naphthenic acid from a synthetic produced water was carried out to evaluate the adsorption capacity of the synthesized nanoadsorbents and to correlate this performance with their physicochemical properties, especially specific surface area, surface chemistry, and zeta potential. In general, aliphatic organic molecules are more readily adsorbed than those with aromatic rings (Samrat Alam et al. 2016). For this reason, benzoic acid was chosen as the model compound, as it is considered a recalcitrant acid with lower removability compared to other naphthenic acids (Singh et al. 2020). Thus, adsorbents capable of efficiently removing benzoic acid are expected to be even more effective for the adsorption of other naphthenic acids in produced water.

**Fig. 8:**
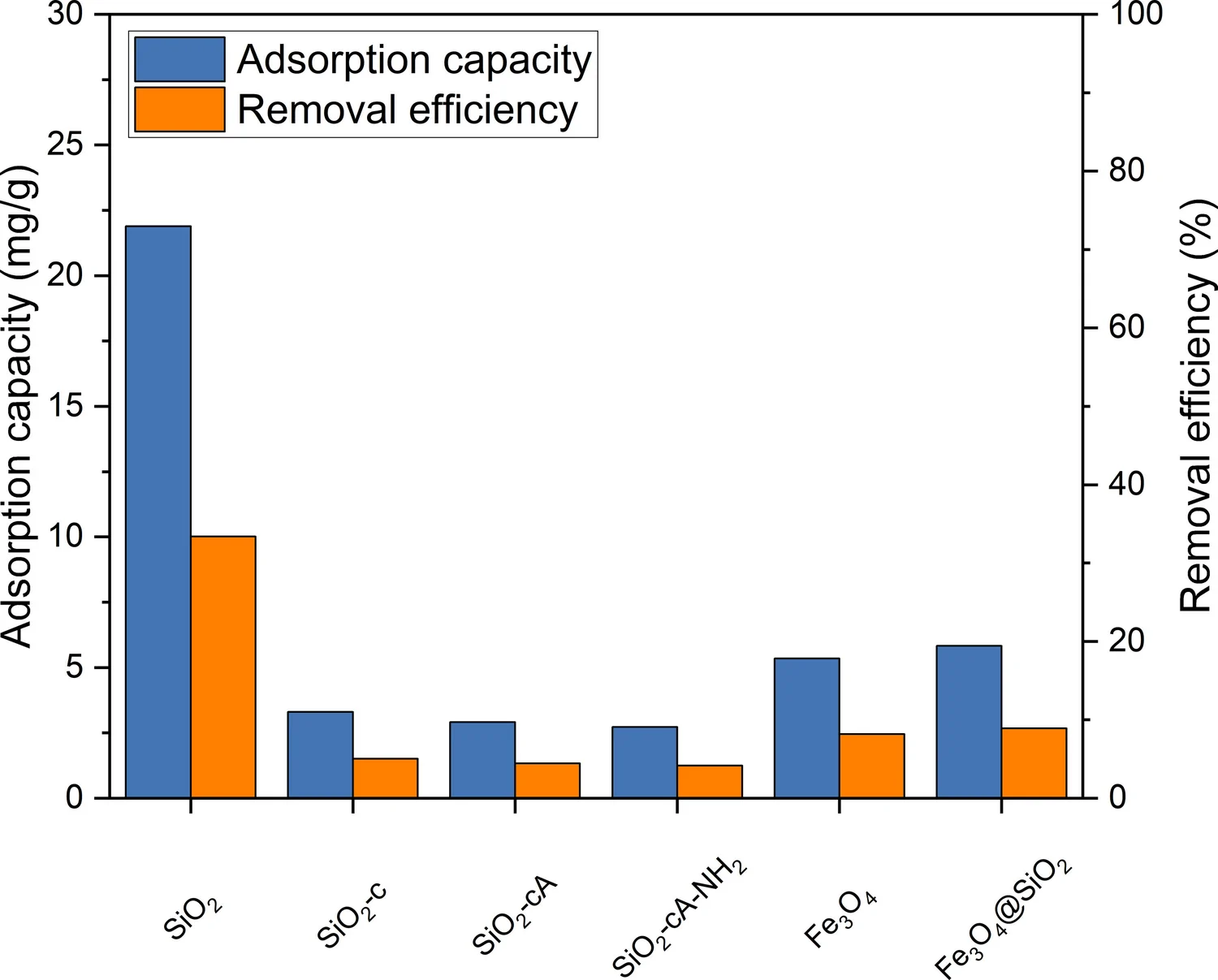
Adsorption capacity and removal efficiency of benzoic acid. Experimental conditions: initial concentration (Ci) = 262 mg/L; temperature = 30 °C; pH = 3; volume = 50 mL; adsorbent mass = 200 mg; contact time = 24 h

Under the tested conditions, the SiO_2_ nanoparticle exhibited the highest adsorption efficiency, with an adsorption capacity of 21.9 mg/g and a benzoic acid removal efficiency of 33.4% (Fig. 8). Moreover, this sample showed a remarkably high removal per surface area, reaching 0.7292 mg/m^2^, which was significantly greater than that of the other samples (Table 5). The remaining nanoparticles displayed adsorption capacities between 2.7 and 5.8 mg/g. These findings are consistent with previous literature. For instance, Singh et al. (2020) investigated benzoic acid adsorption using various biochars and achieved a maximum adsorption capacity of 7.9 mg/g in batch experiments (35 °C, pH 2, d = 2 g/L, Ci = 40 mg/L). Similarly, Strong et al. (2023) used petroleum coke-derived activated carbon to adsorb cyclohexanoacetic acid (pH 8, d = 5 g/L, Ci = 40 mg/L), reporting a capacity of 23 mg/g. Comparable results were also observed by other authors for the adsorption of different naphthenic acids (Iranmanesh et al. 2014; Medeiros et al. 2023b).

**Table 5:** Adsorption capacity, adsorption capacity normalized by surface area, and benzoic acid removal efficiency. Experimental conditions: initial concentration (Ci) = 262 mg/L; temperature = 30 °C; pH = 3; volume = 50 mL; adsorbent mass = 200 mg; contact time = 24 h

Sample	*q* (mg/g)	*R* (%)	*As*_*BET*_ (m^2^/g)	*q*/*As*_*BET*_ (mg/m^2^)
SiO_2_	21.9	33.4	30	0.7296
SiO_2_-c	3.3	5.0	835	0.0040
SiO_2_-cA	2.9	4.5	319	0.0091
SiO_2_-cA-NH_2_	2.7	4.2	118	0.0231
Fe_3_O_4_	5.3	8.2	39	0.1371
Fe_3_O_4_@SiO_2_	5.8	8.9	115	0.0507

In comparison with previous literature (Table 6), the nanoadsorbents synthesized in this study offer several advantages. Primarily, there is a lack of research employing nanoadsorbents for the adsorption of naphthenic acids or WSO from produced water. The reduction in adsorbent size provides an increased specific surface area, minimizing the required dosage for treatment under similar conditions. For example, Medeiros et al. (2023b) reported a notably higher dosage requirement, up to 300 g/L, to achieve a significant reduction in the concentration of model naphthenic acids. Furthermore, SiO_2_ demonstrates superior performance in benzoic acid adsorption compared to other materials. As previously mentioned, it is approximately 3 times more efficient than the rice straw biochar utilized by Strong et al. (2023). Finally, when comparing studies conducted at pH 8.0–8.5 (close to that of produced water in real-world operations), the SiO_2_ nanoadsorbent stands out with a higher adsorption capacity than carbon-based materials (de Farias et al. 2024) and peat-mineral mix or Pleistocene fluvial sands (Medeiros et al. 2023b). An adsorption capacity comparable to sludge-based activated biochar, used in a study for real oil sands process water treatment (Medeiros et al. 2023a), was also achieved. The performance of SiO_2_ under these specific conditions will be detailed in the subsequent sections.

**Table 6:** Performance comparison of various adsorbents for WSO removal from produced water over the last decade

Adsorbent	Model acid or produced water	Adsorption capacity (mg/g)	Conditions				Reference
			Ci (mg/L)	Dosage (g/L)	pH	***T*** (°C)	
SiO_2_	Benzoic acid	21.9	262	4	3	30	Present study
		7.3	100				
		4.2	150		8		
	Cyclohexaneacetic acid	10.3	100		3		
	Naphthenic acid (commercial mixture)	21	178		8		
SiO_2_-c	Benzoic acid	3.3	262		3		
SiO_2_-cA		2.9					
SiO_2_-cA-NH_2_		2.7					
Fe_3_O_4_		5.3					
Fe_3_O_4_@SiO_2_		5.8					
Bleached cellulose biochar	Cyclohexanecarboxylic acid	29.02	30	1	4	25	Machado et al. (2025)
Carbon-based materials	Cyclohexanecarboxylic acid	3.71	12.81	1	8	25	de Farias et al. (2024)
Coconut husk activated charcoal	Cyclohexanecarboxylic acid	114.77	206.5	4	4	50	Costa Louzada et al. (2023)
	Valeric acid	53.85					
	Cyclohexaneacetic acid	122.33					
	Oilfield produced water	13.33	206.5	4	5	25	
Peat-mineral mix	Decanoic acid	16.8	20—100	3 or 5	8.5	20	Medeiros et al. (2023b)
	5-Phenylvaleric acid	10					
	Oil sands process water	1.9	46.3	5 or 50	-	20	
Pleistocene fluvial sands	Decanoic acid	0.5	20—100	300	8.5	20	
	5-Phenylvaleric acid	0.476					
	Oil sand process water	0.127	46.3	100	-	20	
Activated carbon from petroleum coke	Diphenylacetic acid	62	40	5	8	-	Strong et al. (2023)
	Cyclohexaneacetic acid	23					
	Heptanoic acid	27					
Sludge-based activated biochar	Oil sand process water	26.6	61.8	2	8.5	20	Medeiros et al. (2023a)
Mesoporous zeolite Socony Mobil-5 (ZSM-5)	Cyclohexanecarboxylic acid	195	500	3	4	25	Migliorin et al. (2022)
Bovine bone activated charcoal	1,4-Cyclohexanedicarboxylic acid	496.39	1000	1.67	-	30	Lopes et al. (2022)
*Orbignya phalerata* shell activated carbon	1,4-Cyclohexanedicarboxylic acid	417	1000	0.067—2.67	-	25	Mônaco et al. (2020)
Rice straw biochar	Benzoic acid	7.1	5—40	2	2	35	Singh et al. (2020)
Activated petroleum coke	2-Naphthoic acid	451	40	-	6	23	Niasar et al. (2019)
	Diphenylacetic acid	362					
	Cyclohexanepentanoic acid	320					
Granular activated carbon	Oil sand process water	9.5	19.15	2	-	21	Islam et al. (2018)
Commercial activated carbon	2-Naphthoic acid	305	40	0.4	4	23	Niasar et al. (2016)
	Diphenylacetic acid	300					
	1,4-Cyclohexanedicarboxylic acid	430					
Petroleum coke activated carbon	2-Naphthoic acid	776					
	Diphenylacetic acid	624					
	1,4-Cyclohexanedicarboxylic acid	566					
Granular activated carbon	2-Naphthoic acid	357.9	40	0.057—0.580	4	23	Martinez-Iglesias et al. (2015)
	Diphenylacetic acid	317.7					
	1,4-Cyclohexanedicarboxylic acid	452.6					

Despite the low specific surface area of the SiO_2_ sample, its performance in the adsorption of benzoic acid was superior to that of the other nanoparticles. This result may be related to the sample’s hydrophobic surface characteristics. At pH 3, the condition applied in the experiment, SiO_2_ carries a positive surface charge (pH < pI), while benzoic acid remains in its molecular form (pH < pKa). In such conditions, the adsorption is likely driven by hydrophobic interactions, including Van der Waals forces, between the hydrophobic adsorbent surface and the naphthenic acid molecules (Singh et al. 2020). Comparable findings were observed by Niasar et al. (2016), who investigated the adsorption of different naphthenic acids onto activated carbon, and by Hendges et al. (2021), who studied the removal of cyclohexanecarboxylic acid using commercial adsorbents.

Although magnetic nanoparticles offer the advantage of easy recovery after adsorption via electromagnetic field application (Lata and Samadder 2016), the Fe_3_O_4_ and Fe_3_O_4_@SiO_2_ samples showed lower adsorption performance than the SiO_2_ sample. Nonetheless, they outperformed the other silica-based samples, highlighting their potential as effective adsorbents.

Due to the higher specific surface area of the SiO_2_-c, SiO_2_-cA, and SiO_2_-cA-NH_2_ samples compared to SiO_2_, a greater adsorption capacity would be expected for these nanoparticles. These materials exhibit diverse surface chemistries, including the presence of CTAB (SiO_2_), varying concentrations of surface hydroxyl groups (SiO_2_-c and SiO_2_-cA), and aminopropyl groups (SiO_2_-cA-NH_2_). All nanoparticles showed a positive surface charge at the pH used in the adsorption test, except for SiO_2_-c, which was close to its isoelectric point. Therefore, it can be concluded that the type of surface functional group plays a significant role in determining adsorption capacity.

For the SiO_2_ nanoparticles, the CTAB molecules adsorbed on their surface contain a quaternary ammonium headgroup and a hydrophobic C16 alkyl chain, resulting in hydrophobic surface domains due to the outward orientation of the surfactant’s alkyl chains. These hydrophobic groups play a key role in the interaction with the naphthenic acid, which, being in its molecular form, also exhibits hydrophobic characteristics. Therefore, it can be concluded that the surface modification with CTAB is the primary driver for the successful removal of benzoic acid by SiO_2_, and that hydrophilic adsorbents would not be as effective.

In addition, the surface functionalization of silica nanoparticles with CTAB or amino groups was intended to raise the isoelectric point. This modification was designed to facilitate electrostatic interactions between the positively charged surface of the nanoparticles and the deprotonated naphthenic acid species. Nevertheless, at pH 3, naphthenic acid is in its neutral molecular state. Thus, adsorption cannot occur via electrostatic interactions. Alternative pH levels were assessed and are discussed in the next section, confirming that the highest adsorption efficiency occurred at pH 3, which was selected for evaluating the adsorption capacity of all nanoadsorbents.

### Influence of acid type and pH on the adsorption process

A 2 × 3 factorial design of experiments (DOE) was employed to evaluate the effects of acid type and pH on adsorption efficiency when SiO_2_ was used as the nanoadsorbent. The regression model included a linear term for the type of acid, linear and quadratic terms for pH, and an interaction term (pH × acid type). The model was globally significant (*F* = 40.5, *p* < 0.001) and accounted for 97.0% of the response variability. The adjusted model for adsorption efficiency is expressed in Eq. 4, where *y* is the adsorption efficiency and *x*_*1*_ and *x*_*2*_ are the coded values for acid type and pH, respectively.

4 $$y=12.1267+3.4567 {x}_{1}-15.0935 {x}_{2}-1.1485{x}_{1}{x}_{2}+11.3598 {x}_{2}^{2}$$

**Fig. 9:**
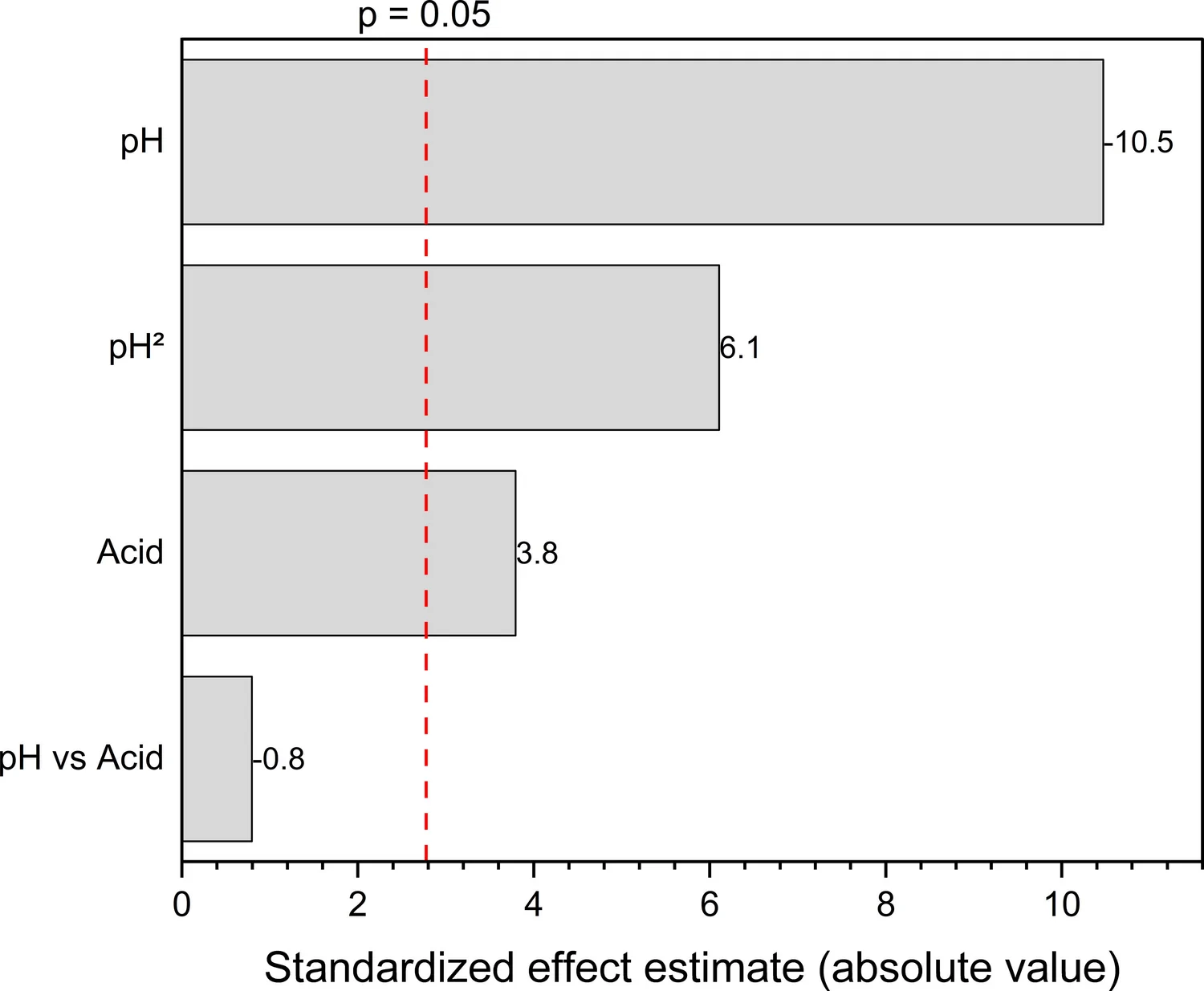
Pareto chart for the removal efficiency

The significant parameters were identified using the *p*-values from the adjusted equation and the standardized Pareto chart, as illustrated in Fig. 9. According to both analyses, the linear term of pH was negative and significant (*p* < 0.05), indicating that, at the studied range, adsorption efficiency increases as pH decreases. The quadratic term for pH was included to evaluate the curvature of the model and was also significant (*p* < 0.05), suggesting that the intermediate pH has a negative effect on the adsorption. This behavior is consistent with the transition between the ionized and protonated forms of the acids and the negative and positive charges of the nanoadsorbent. Additionally, the significant linear term of acid type (*p* < 0.05) indicates that the type of acid statistically influences the adsorption efficiency. Benzoic acid exhibited greater resistance toward removal compared to cyclohexaneacetic acid under the same conditions. This difference can be attributed to the chemical structure and hydrophobicity of the model acids. Cyclohexaneacetic acid is more hydrophobic (pKa = 4.94, log Kow = 2.85) (Wishart et al. 2022) and interacts more strongly with the hydrophobic SiO_2_ surface than the smaller, aromatic benzoic acid (pKa = 4.2, log Kow = 1.87) (Lee and Chen 2009; Karimova et al. 2020). Finally, the interaction term was not statistically significant (*p* > 0.05). Therefore, when SiO_2_ is used as an adsorbent, it is expected that the reduction of pH will increase the adsorption efficiency, regardless of the specific naphthenic acid present.

**Fig. 10:**
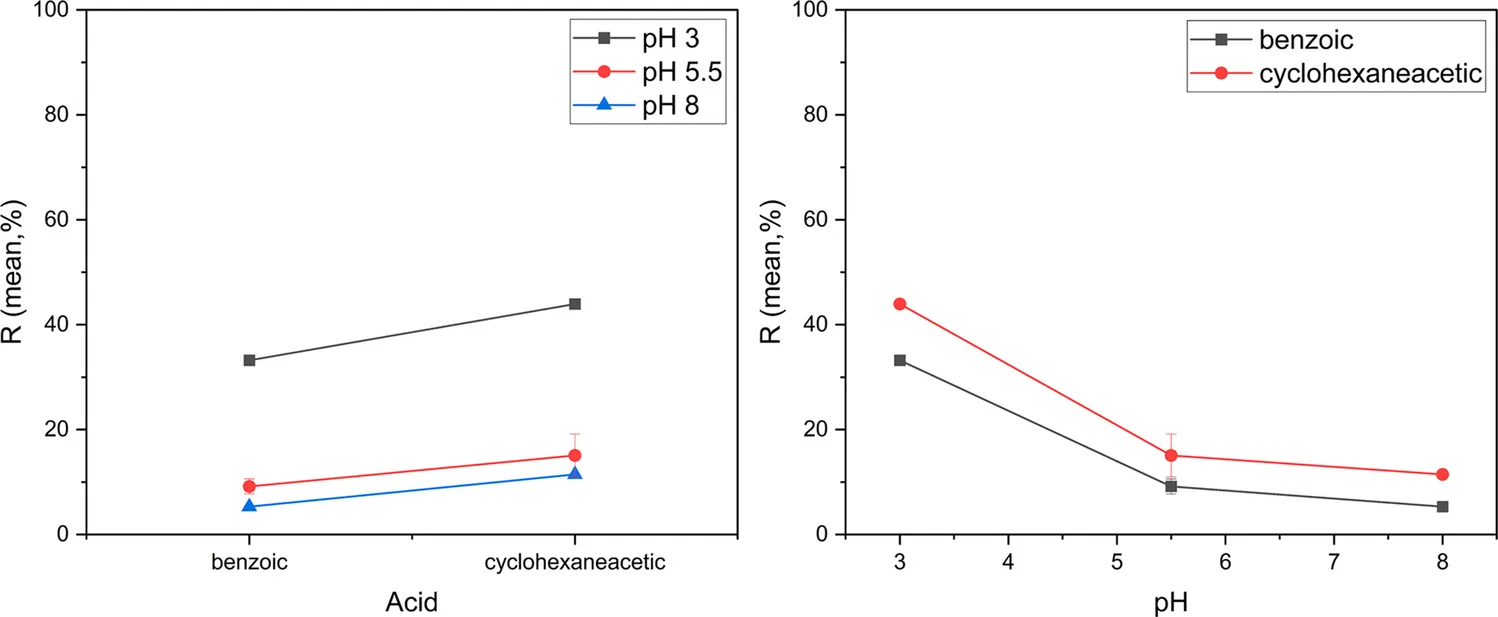
Interaction plots. There is no interaction between factors

Interaction plots (Fig. 10) further evaluate the influence of pH and acid type on the adsorption process. Consistent with the Pareto chart analysis, the parallel lines of the plots confirm the absence of interaction effects between the two variables. It can also be concluded that there is an inverse relationship between pH and removal efficiency. At pH 3, below both acids’ pKa values and the SiO_2_ isoelectric point (pI = 6.3), both acids are protonated, and the nanoadsorbent is positively charged. Under these conditions, the interactions between the model naphthenic acids and the nanoadsorbent are likely to be due to hydrophobic interactions, as concluded in the previous section. Notably, at pKa < pH < pI (e.g., pH 5.5), where the acids are deprotonated and negatively charged, and the adsorbent remains positive, adsorption efficiency decreases relative to pH 3. These findings suggest that hydrophobic interactions predominate over electrostatic interactions within the adsorption process. A similar result was obtained by Hendges et al. (2021), who evaluated the adsorption of cyclohexanecarboxylic acid from synthetic produced water. According to the authors’ conclusions, the highest adsorption efficiency is achieved at pH values below the pKa of the naphthenic acid. Conversely, at pH 8, which is close to the pH of real produced water, a significant decrease in adsorption efficiency occurs.

Additionally, the Tukey test indicated that the difference between the means at pH 5.5 and pH 8 is not statistically significant within a 95% confidence interval. Consequently, it can be inferred that the treatment of produced waters within the pH 5.5–8 range, typical for oilfield produced waters (Rajbongshi and Gogoi 2024), will result in consistent adsorption performance. This lack of sensitivity to pH fluctuations within this specific interval enhances the reliability and predictability of the process for industrial applications.

### Adsorption of a representative multicomponent synthetic produced water

**Fig. 11:**
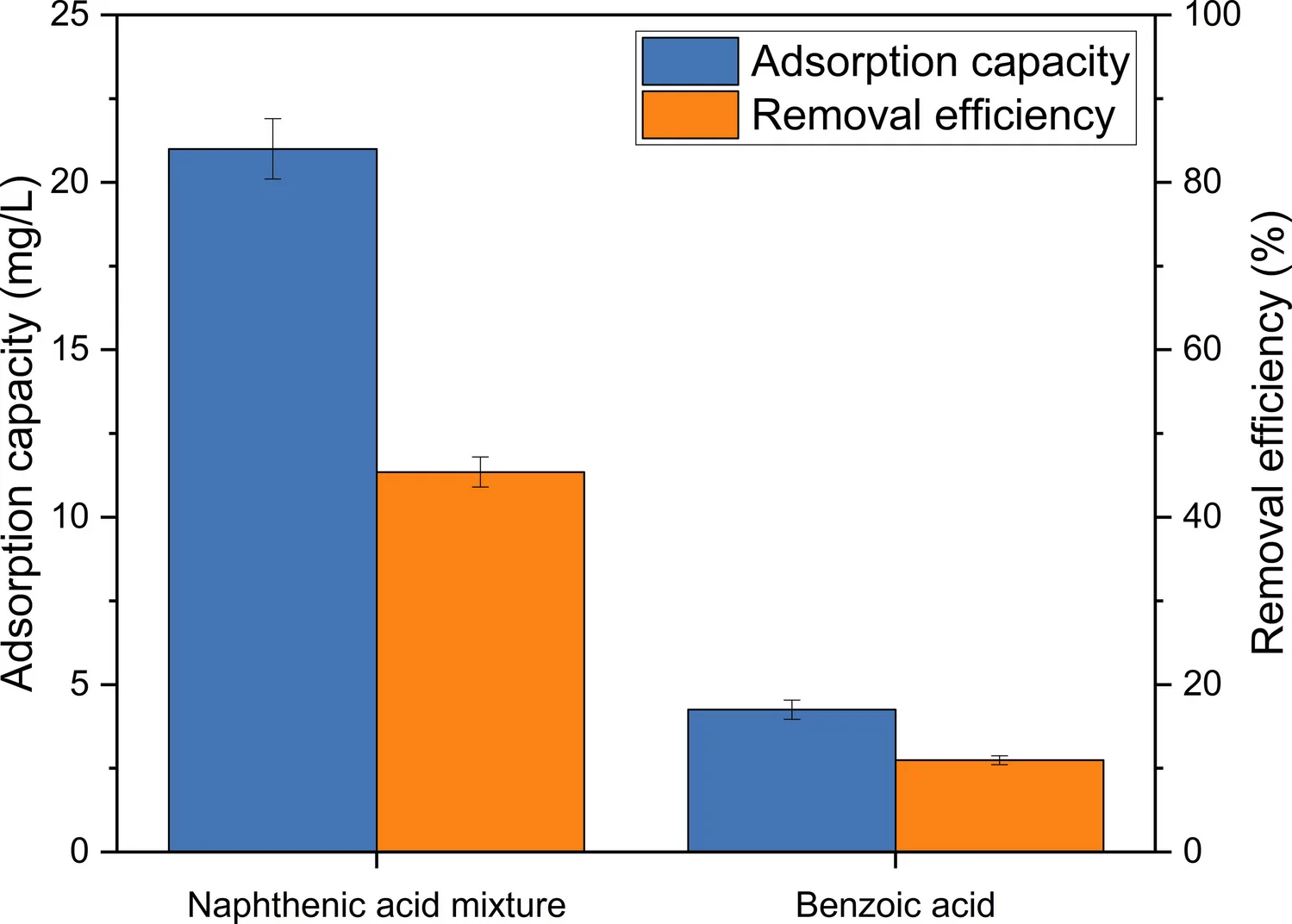
Adsorption capacity and removal efficiency of the naphthenic acid mixture and benzoic acid. Experimental conditions: initial concentration (Ci) = 178 and 150 mg/L, respectively; temperature = 30 °C; pH = 3; volume = 50 mL; adsorbent mass = 200 mg; contact time = 24 h

Adsorbent performance was further evaluated in a representative multicomponent synthetic produced water at pH 8, to reflect real-world conditions. The SiO_2_ nanoadsorbent performed significantly better in the complex matrix than in the benzoic acid model. Adsorption capacity for the multicomponent matrix was 21.0 mg/g (45.4% removal), more than five times higher than the 4.2 mg/g (11.0%) observed for benzoic acid (Fig. 11). This improvement suggests that several acids within the naphthenic mixture have higher affinities for the adsorbent than benzoic acid, even at higher pH. These results indicate that SiO_2_ and the other synthesized nanoadsorbents will likely exhibit even superior removal of WSO from real oilfield produced water compared to the results obtained with individual acid models.

## Conclusions

In this study, seven types of silica and magnetic nanoparticles were successfully synthesized, modified, and thoroughly characterized to assess their potential as nanoadsorbents for the removal of naphthenic acids from produced water. Functionalization of SiO_2_ with CTAB was advantageous, as this nanoparticle exhibited a hydrophobic surface that enhanced adsorption performance. Calcination effectively eliminated CTAB but also led to the degradation of surface silanol groups, which are essential for further functionalization. To address this, an activation step was introduced to regenerate –OH groups (SiO_2_-cA), thereby enabling successful functionalization of sample SiO_2_-cA-NH_2_ with APTMS. Magnetite nanoparticles (Fe_3_O_4_) were synthesized and coated with a silica layer, forming core–shell magnetic nanoparticles (Fe_3_O_4_@SiO_2_), which were then functionalized with APTMS (Fe_3_O_4_@SiO_2_-NH_2_). The encapsulation process led to an increase in nanoparticle size but increased the specific surface area due to the porous structure of the silica shell.

Adsorption experiments conducted with benzoic acid as a model compound revealed that the SiO_2_ nanoparticle exhibited the highest performance, with an adsorption capacity of 21.9 mg/g, corresponding to 33.4% removal of benzoic acid. This result is associated with the presence of CTAB and it can be inferred that the adsorption of benzoic acid occurred predominantly through hydrophobic interactions, such as Van der Waals forces. Even though the SiO_2_ nanoparticle demonstrated the highest adsorption efficiency, the other nanoparticles should not be excluded as potential nanoadsorbents. Especially in the case of magnetic and functionalized nanoparticles, their combination of selective adsorption and convenient recovery post-saturation highlights their potential for practical applications.

The influence of acid type and pH on adsorption was evaluated, and a significant regression model (*p* < 0.001) identified pH as the dominant variable, with an inverse relationship between pH and removal efficiency. Mechanistically, it was demonstrated that hydrophobic interactions predominate over electrostatic attraction, as evidenced by higher adsorption at pH 3 compared to the deprotonated state at pH 5.5. Furthermore, the statistical similarity in performance across the pH 5.5–8 range confirms the robustness of the SiO_2_ nanoadsorbent for oilfield applications, where fluctuations in the pH are common. Finally, the superior removal capacity observed in multicomponent matrices 21.0 mg/g relative to single-component models (4.2 mg/g) highlights the high potential of these nanoadsorbents for the treatment of complex, real-world produced waters.

Overall, the findings underscore the critical role of surface chemistry in determining adsorption efficiency and highlight the trade-off between high adsorption capacity and practical recoverability. Future studies should extend these investigations to real produced water and evaluate the regeneration capacity and long-term stability of the adsorbents over multiple cycles. In addition, the application of kinetic and thermodynamic models may contribute to a deeper understanding of the adsorption mechanisms involved in this process and support the development of scalable nanomaterial-based polishing processes for enhanced produced water treatment.

## Data Availability

The datasets generated and analyzed during the current study are available from the corresponding author on reasonable request.
